# Development and pilot implementation of a locally developed Trauma Registry: lessons learnt in a low-income country

**DOI:** 10.1186/1471-227X-13-4

**Published:** 2013-03-21

**Authors:** Amber Mehmood, Junaid Abdul Razzak, Sarah Kabir, Ellen J MacKenzie, Adnan A Hyder

**Affiliations:** 1 Department of Emergency Medicine, Aga Khan University, Karachi, Pakistan; 2 Bloomberg School of Public Health, John Hopkins University, Baltimore, USA; 3 Johns Hopkins International Injury Research Unit, Bloomberg School of Public Health, Baltimore, USA

**Keywords:** Trauma registry, Pakistan, Injury, Surveillance, Outcome

## Abstract

**Background:**

Trauma registries (TRs) play an integral role in the assessment of trauma care quality. TRs are still uncommon in developing countries owing to awareness and cost. We present a case study of development and pilot implementation of “Karachi Trauma Registry” (KITR), using existing medical records at a tertiary-care hospital of Karachi, Pakistan to present results of initial data and describe its process of implementation.

**Methods:**

KITR is a locally developed, customized, electronic trauma registry based on open source software designed by local software developers in Karachi. Data for KITR was collected from November 2010 to January 2011. All patients presenting to the Emergency Department (ED) of the Aga Khan University Hospital (AKUH) with a diagnosis of injury as defined in ICD-9 CM were included. There was no direct contact with patients or health care providers for data collection. Basic demographics, injury details, event detail, injury severity and outcome were recorded. Data was entered in the KITR and reports were generated.

**Results:**

Complete data of 542 patients were entered and analysed. The mean age of patients was 27 years, and 72.5% were males. About 87% of patients had sustained blunt injury. Falls and motor vehicle crashes were the most common mechanisms of injury. Head and face, followed by the extremities, were the most frequently injured anatomical regions. The mean Injury Severity Score (ISS) was 4.99 and there were 8 deaths. The most common missing variables in the medical records were ethnicity, ED notification prior to transfer, and pre-hospital IV fluids. Average time to review each chart was 14.5 minutes and entry into the electronic registry required 15 minutes.

**Conclusion:**

Using existing medical records, we were able to enter data on most variables including mechanism of injuries, burden of severe injuries and quality indicators such as length of stay in ED, injury to arrival delay, as well as generate injury severity and survival probability but missed information such as ethnicity, ED notification. To make the data collection process more effective, we propose provider based data collection or making a standardized data collection tool a part of medical records.

## Background

Trauma registries (TRs) are databases used to monitor and enhance the quality of trauma care and public health programs related to injury prevention and research [1-3]. The scope of a particular TR determines the amount of information captured through them and may vary from a “minimal dataset” collected in emergency departments (ED) to a “comprehensive dataset” with information from pre-hospital care to rehabilitation [4-8]. While maintaining TR is a requirement of many trauma systems, standardization of variables is important to ensure outcome comparison in terms of patient and injury characteristic [1,3,7,9]. Trauma registries are well established in in many high-income countries (HIC) such as United States; have been used to promote injury prevention, change policies and to evaluate trauma system effectiveness [10]. In many instances, the registries are guided through the American College of Surgeons guidelines for selection of data points [2,7,11].

Ninety per cent of trauma- and injury-related deaths and disabilities occur in low-and middle-income countries (LMICs) [12]. A significant number of these deaths can be averted through improvement in trauma care in these countries [6,13-16]. However, because information on injuries and trauma from LMICs is most often based on routine health surveys, surveillance reports, police data and hospital-based case series, information about the process and quality of trauma care or clinical outcomes is lacking [8,12,17-20]. Limited useful information on trauma care in LMICs underscores the importance of TRs in these settings. Examples of successful implementation of trauma registries in LMICs are also uncommon due to the cost of obtaining and maintaining a TR [1,3,12,16].

Currently available commercial TRs such as Collector©, Trauma One© and NTRACS© are expensive products. For instance, Collector© which has over 1500 clients in 10 countries, costs about 7500 USD for application and 2500 USD for yearly license. The cost of training and updates are in addition to maintenance, which makes it and other commercial products inaccessible for many LMICs. TRs in many of the developing countries are under-developed, incomplete and used for surveillance purposes [3]. A locally developed electronic trauma registry is thus needed to assess injury adjusted trauma outcomes and to test this software in a hospital setting.

The objective of this study is to describe the structure, process of development and pilot implementation of a locally developed, electronic trauma registry – the “Karachi Trauma Registry” (KITR) - from Karachi, Pakistan using existing medical records. We also share the lessons learnt during the implementation in a low income country.

## Methods

### The development of electronic trauma registry

**Figure 1:**
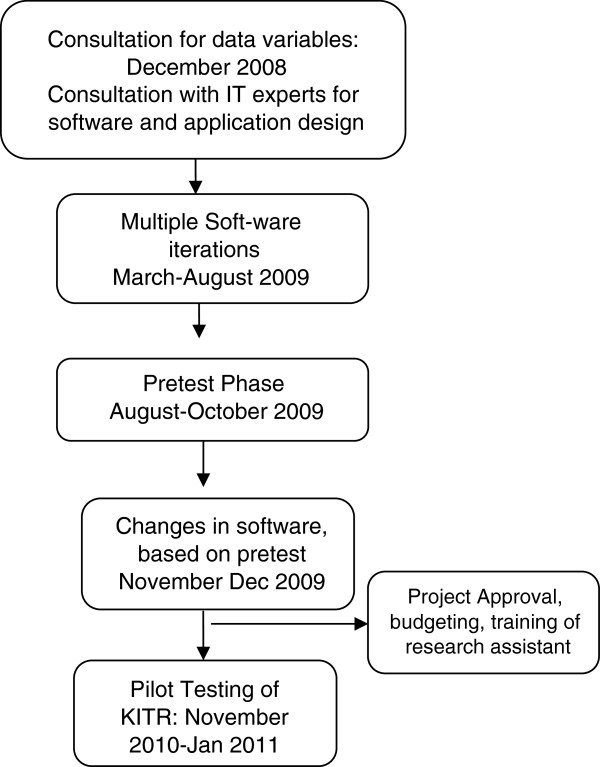
Development and implementation process Karachi Trauma Registry.

The development of electronic registry was a four step process (Figure 1) which was followed by pilot testing. The development began in December 2008 with finalization of variables by a team consisting of a general surgeon, emergency physician, and public health professionals with special interest in trauma outcome research. In the next step, the IT experts were consulted for software and application design. The variables were organized for calculation of survival probability as well as ensuring that all the stages in-hospital treatment were recorded with date, time and interventions. The development of the electronic registry (KITR) required multiple iterations between March-August 2009, and open source softwares were used during the programming. The first software version was pre-tested on 120 trauma cases in August- October 2009 to check the data entry, any errors, collation of data and back-hand calculators. Based on the findings of pre-test, further modifications were carried out. The final product was a Windows-XP^®^ based software which could be installed as a stand-alone database system on PC and required Pentium III or higher processor, with a hard disk storage capacity (RAM) of at least 1 GB. The registry was based on SQL Server 2005^®^ and is also supported by SQL Server express^®^, which provides storage, processing and controlled access of data. KITR required dot net (.Net) Framework 3.0^®^ and Microsoft Excel 2007^®^ for pivot table analysis but does not require an internet connection.

### Data handling and derivation of trauma indices

**Figure 2:**
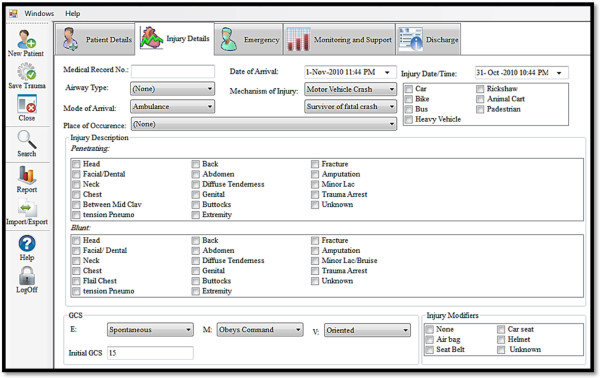
Snapshot of KITR with dropdown menus and tabs.

To facilitate data entry, separate tabs for recording patient demographics, injury details, emergency evaluation, treatment, in-hospital course and discharge details were provided (Figure 2). Several dropdown menus and a checklist were provided to minimize free text entry as much as possible. The built-in spread sheets and calculators helped store, collate and analyse data. Details of insurance or payer information were, however, not a part of the registry. The software was password protected and security of the database was ensured by encryption at the server, which was also login sensitive. The KITR used International Statistical Classification of Diseases and Related Health Problems (ICD 9 - CM) and Abbreviated Injury Scaling (AIS) 2005 [21] for standardization of definitions and injury scaling. The registry was capable of generating different trauma scores (Glasgow Coma Scale, Revised Trauma Score, Injury Severity Score) and probability of survival (Trauma Injury Severity Score - TRISS) score [22].

### Pilot implementation

The pilot study was conducted over a three-month period (November 2010 to January 2011) in the ED of the AKUH in Karachi, Pakistan.

### Setting

AKUH is a 650- bedded tertiary referral centre, with 50,000 annual ED visits and training programs in Emergency Medicine and Trauma Surgery among others. The hospital has a 24-hours on-call trauma team comprising of Emergency physicians and residents from general surgery, orthopaedics, anaesthesia and neurosurgery. Some of the health information is available as electronic records such as triage list, admissions, laboratory, radiology, discharge summaries etc. while the history and physical examination and progress notes are manually written in the files.

#### Case definition

All trauma patients presenting to the ED with history of trauma within 24 hours, or transferred from other hospitals and coded as International Classification of Disease (ICD) injury codes (ICD-9-CM 800–959.9) were included in this study. Isolated hip fractures and dead-on-arrival trauma patients were excluded. Since AIS and TRISS scores cannot be derived for poisoning, these cases were also excluded. The cases included both genders and all age groups.

#### Data sources

The data sources included medical records; doctors’ and nurses’ notes; laboratory, radiology, and operative reports and discharge summaries. Daily report of ED visits with age, primary complaint and disposition was obtained from the electronic health information system. The triage, admission, and ED discharge list were utilized to capture patients with injuries.

#### Data collection and entry

For this pilot study the medical records of trauma patients were reviewed by a research assistant trained in medical chart abstraction, ICD-9 injury codes, AIS and injury severity scoring. A form was used for data collection, which did not involve direct contact with patients or their attendants. The information consisted of details about the patient’s demographics, injury event and mechanism, physiological parameters, investigations, severity of injury, operative and non-operative procedures, complications, discharge capacity and follow-up until patient’s discharge from the hospital or death of the patient.

Random checks of the medical charts were performed by the principal investigator for accuracy and completeness of data collection during the study to compare the actual information and that on the hard copies. All ICD codes and AIS scores were cross checked prior to data entry by the PI and errors were corrected. All Electronic records were cross checked for accuracy and discrepancies noted, however once data entry had taken place, no items were changed, modified, or corrected. Missing or incorrect items were listed as shown in the Table 1.

**Table 1:** Item completion and errors

**S. No.**	**All patients (n = 542)**	**Data completion (in percent)**	**Errors identified**
1	Age	100	1
2	Gender	100	0
3	Unique ID	100	0
4	Ethnicity	100	1
5	City ID	100	0
6	Place of occurrence	97.23	0
7	Mode of arrival	100	1
8	Date of arrival	100	0
9	Injury time	100	0
10	Injury mechanism	100	0
11	Transfer in	97.6	0
12	Trauma code activated	96.9	0
13	ICD-9 code	100	6
14	AIS code	98.3	9
15	Injury severity Score	98.3	
16	Revised trauma score	100	3
17	TRISS	97.9	
100	ED exit time	100	0
19	Discharge date	100	0
20	Final outcome	100	0
21	Discharge capacity	99.8	1
	**Where applicable (n):**		
22	Safety equipment (179)	97.7	0
23	Operative procedures (137)	100	0
24	In-hospital blood products (53)	100	0
25	Radiological imaging in ED (391)	100	1
26	Labs reports in ED (273)	95.9	2
27	ED notified before transfer in (92)	30.4	

#### Reports

Basic frequency tables were produced on the number of admissions, demographics, mechanism of injuries, ICD -9 coding of injuries, discharge disposition, length of stay, probability of survival and actual survival.

The pilot study protocol was approved by the Ethics Review Committee of the Aga Khan University.

## Results

### Cost of KITR development and pilot testing

The development of KITR from concept to operational software took 23 months. The estimated cost for the development of the software was USD 9,600. This included the time of investigators (54% of estimated cost), the cost of software development (16% of estimated), and implementation cost (30% of estimated). The actual cost incurred was the implementation cost in the form of stipends of research assistant and miscellaneous expenditure.

### Case ascertainment and item completion

Triage and admission/discharge list indicated 946 cases; however, number of records within the case definition was 732 during the study period. The number of cases used for the registry was 542 (74%); reasons included non-availability of charts for review (n = 176), patients still receiving care in hospital during study period (n = 3) or insufficient documentation of injuries to assign AIS scores (n = 10). Table 1 shows item completion and errors. Some variables which were a part of the registry, were not documented in the medical charts; for instance ethnicity (95%), the amount of IV fluids administered in pre-hospital phase (94%), Safety Equipment (81%) and ED notification prior to arrival of patients (90%). These undocumented variables are entered as “unknown” in the KITR. For those patients who were transferred in, ED was notified in only 8.6% cases. Total 25 data points were found as erroneous. Errors in AIS and ICD included nine AIS scores (1.7% of all cases) and six ICD codes (1% of all cases) were corrected prior to data entry and other 10 items (Table 1) were recognized as wrong data entry at the time of verification of electronic data.

### Time burden

The mean time for data retrieval and entry was 29.5 minutes (range 15–50 minutes) per case. Time for data abstraction and hard copy questionnaire completion was 14.5 minutes (range: 8–20 minutes) while the mean time for data entry was 15 minutes (range: 7–30 minutes) based on the number of entries. (Total time 29.5 minutes and a range of 15–50 minutes) This time burden excludes the time taken for double checking the records or data entry in the registry.

### Patient characteristics and injury mechanism

Table 2 gives demographic details and distribution of injury severity scores (ISS). Mean age of the victims were 27 years (range: 1–89 years) and males represented a higher proportion of recorded cases in all age groups (n = 394; 72.6%). The most common mechanisms of injury were fall (37%), motor vehicle crash (33%), and gunshot injuries (7%). Miscellaneous injuries (16%) included sports injuries, assault with blunt object, bites and occupational injuries.

**Table 2:** Demographic details of captured cases in KITR according to ISS

**Variables**	**Injury severity score (ISS)**		**Total**
	**≤9**	**>9**	
**Gender**			
Male	307	87	394
Female	140	8	148
**Age Group**			
under 15	155	9	164
15 - 29	123	33	156
30 - 44	34	24	58
45 - 64	51	24	75
more than 64	24	5	29
**Total**	447	95	542

### Injury severity and survival analysis

**Figure 3:**
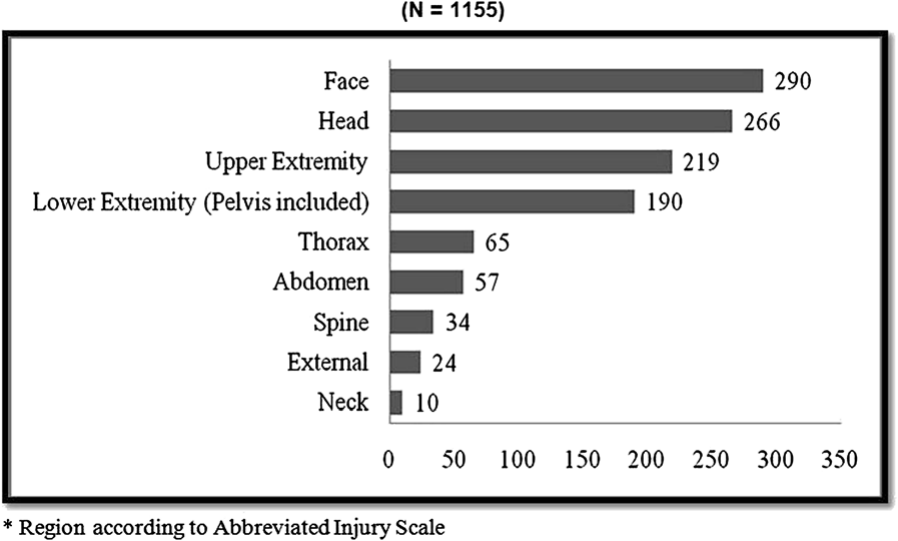
Frequency of injuries according to anatomical region* (N = 1155). * Region according to Abbreviated Injury Scale.

Many patients presented with multiple injuries located in more than one anatomical region; therefore 1155 injuries were recorded in KITR from 542 cases. The most common injuries included head, face and upper extremity injuries (Figure 3).

As shown in Table 2, 82% of the patients in our sample had an Injury Severity Score of ≤9 categorized as mild, 9% had ISS: 9–15 classified as moderate injuries, 7% had ISS between 16–25, and only 2% had ISS of >25 representing critical injuries. 2.6% of patients had a probability of survival of less than 50% (Table 3). Eight patients (1.47%) died; five of those who died had a probability of survival of <50%. Disability at the time of discharge was recorded as per clinicians’ assessment in the medical charts. More than half of the patients (n = 287) had no disability at the time of discharge from the hospital, 245 (45.2%) had temporary disability, and 10 (1.84%) had permanent disability at the time of discharge.

**Table 3:** Summary of patient outcomes (n = 542) from pilot test of KITR

**Outcomes**	**Score**
**Mean Injury severity score (ISS)**	4.99 (Range 0–38)
Mean Trauma/ Injury severity score (TRISS)*	96.62%
Number of patients with TRISS ≤ 50	14
Number of patients with TRISS > 50	528
Expired	8
**Total time in ED (in hours)**	**Number of patients (%)**
Less than 1	72
From 1 -8	363
From 8 – 24	71
More than 24	36
**Injury to Hospital Time (in hours)**	**Number of patients (%)**
Less than 4	441
From 4 -8	43
From 8 – 24	50
More than 24	8

### Quality indicators

The registry was capable of generating quality indicators, such as pre-hospital delay, ED length of stay, length of stay in hospital, disposition from ED as well as predicted and actual survival. Although pre-hospital time in 81% of cases was less than 4 hours (range: 10 minutes to 28 hours), the large variability of pre-hospital time can be attributed to inter-facility transfers. Over 80% of patients were either transferred to in-patient units or discharged from the ED in ≤ 8 hours.

## Discussion

This paper describes the three main steps for trauma registry implementation in a developing country; a- the process of development of the registry; b- affordability of its development and implementation and c- the challenges of the implementation of the software. The team of trauma experts and software developers took almost 2 years with a direct cost of USD: 9,600 to develop a functional trauma registry. The most critical test of the success of the effort was in the implementation of the registry in a real hospital based patient care scenario.

There is limited literature on TRs in developing countries [23-25]. Kampala Trauma Registry was developed to establish an injury surveillance system in Uganda [23]. This was a paper based data collection system and attempted to demonstrate the feasibility of a trauma registry in limited resource setting. There was no electronic software and survival analysis was based on Kampala Trauma score (KTS). Similarly, a pilot test of trauma registry was undertaken in Haiti, utilizing a paper form for data collection and Epi Info^®^ for data entry and analysis [24]. The registry variables included mechanism of injury, Glasgow coma score, body region, treatment and investigations but did not anatomical injury scores. The Cape Town Trauma Registry was designed for middle-income setting with a spatial distribution of injury events using GIS mapping, for injury surveillance and control [25]. The above examples are registries with serve as injury surveillance systems and focus on systematic data collection and analysis, with intent to defining issues in implementing a trauma registry in a low income setting. Other examples from LMIC attempted survival outcome comparison with the US Major Trauma Outcome Study [26] or creation of a database to record a particular type of injuries [27]. A recent report from a high-income country in the Middle East described the process of converting a single centre registry into a multicenter database, which is hard to replicate in low-income settings [28].

Similar to other settings, we found four critical success factors for the implementation of trauma registry in our hospital. 1- The fundamental importance of good patient records, patient identification and documentation of all relevant information cannot be overstated. In settings with a paper-based health information system, there would be a need for creating a process of patient identification, data collection and follow-up. The most effective strategy to identify patients post-hoc in our settings was the ED triage where a system of identifying and separating trauma patients was likely to lead to most capture. 2- Training of personnel and availability of technical support to the staff [1,3,7]. 3- A third prerequisite is sustainable funding, which is by far the most common reason for the lack of a long-term implementation plan for a registry [1,3,7,12]. 4- Finally, one of the most important factors which alone can impact these barriers is institutional buy-in from senior hospital management. This provides an impetus for enhancing the quality of trauma care, improves motivation and participation of the care providers, ensures confidentiality of data and protects from medico-legal aspects of providing care to the injured [12,23-25,29].

Data abstraction and case ascertainment from this pilot revealed some important factors which will impact the process of implementation at a larger scale. The coordinator based implementation model did not include direct contact with patients, attendants or health care providers. Potentially it may result in loss of information of some variables which are supposed to be a part of medical records, as in our experience. In those settings where electronic health records are not available, access to medical records can be difficult. The alternative method of provider based data collection may ensure a higher level of completeness but in high volume facilities this could be challenging and more expensive.

### Limitations

The study was done in a single tertiary-care academic institution with a electronic health information system, trauma team and round-the-clock availability of computed tomography (CT) and other diagnostic modalities. This setting may not reflect the reality of all private or public tertiary-care centres in Pakistan or in other developing countries. Wider, multi centre implementation studies would be needed to improve the data collection system and the implementation process.

## Conclusion

KITR is the first electronic trauma registry in Pakistan developed with local resources. This registry was able to generate surveillance data such as mechanism of injuries, burden of severe injuries and quality indicators such as length of stay in ED, injury to arrival delay, injury severity and survival probability. To make the data collection process more effective, provider based data collection or making a standardized data collection tool a part of medical records will be helpful.

## Competing interests

There are no competing interests.

## Authors’ contributions

AM and JAR conceptualized the pilot of the registry and developed its study design. AM was involved directly in the development of the registry. SK helped with data collection, data entry and analysis. AM wrote the first draft and all the revisions. JAR, AAH and EJM provided critical review of the manuscript. All authors read and approved the final manuscript.

## Pre-publication history

The pre-publication history for this paper can be accessed here:

http://www.biomedcentral.com/1471-227X/13/4/prepub
